# Dataset of the binding kinetic rate constants of anti-PCSK9 antibodies obtained using the Biacore T100, ProteOn XPR36, Octet RED384, and IBIS MX96 biosensor platforms

**DOI:** 10.1016/j.dib.2016.07.044

**Published:** 2016-07-27

**Authors:** Danlin Yang, Ajit Singh, Helen Wu, Rachel Kroe-Barrett

**Affiliations:** a Department of Immune Modulation and Biotherapeutics Discovery, Boehringer Ingelheim Pharmaceuticals, Inc., Ridgefield, CT 06877, United States; b The Fu Foundation School of Engineering and Applied Science, Columbia University, New York, United States

**Keywords:** Biacore, ProteOn, Octet, MX96, Antibody–antigen interactions, Optical biosensor

## Abstract

Here we provide data from a head-to-head comparison study using four biosensor platforms: GE Healthcare׳s Biacore T100, Bio-Rad׳s ProteOn XPR36, ForteBio׳s Octet RED384, and Wasatch Microfluidics׳s IBIS MX96. We used these instruments to analyze the binding interactions of a panel of ten high-affinity monoclonal antibodies with their antigen, human proprotein convertase subtilisin kexin type 9 (PCSK9). For each instrument, binding curves obtained at multiple densities of surface antibodies were fit to the 1:1 Langmuir kinetic model, and the association and dissociation rate constants and corresponding affinity constants were calculated. The data supplied in this article accompany the research article entitled, “Comparison of biosensor platforms in the evaluation of high affinity antibody–antigen binding kinetics” (Yang et al., 2016) [1], which further discusses the strengths and weaknesses of each biosensor platform with an emphasis on data consistency, comparability, and operational efficiency.

**Specifications Table**

**Table:** 

Subject area	*Biosensors*
More specific subject area	*Binding kinetic analysis*
Type of data	*Table*
How data was acquired	*GE Healthcare׳s Biacore T100, Bio-Rad׳s ProteOn XPR36, ForteBio׳s Octet RED384, and Wasatch Microfluidics׳s IBIS MX*96
Data format	*Analyzed*
Experimental factors	*Antibodies were captured or immobilized onto the sensor surface at multiple densities, and the antigen (hu PCSK9) was flowed over the antibody surface at titrating concentrations.*
Experimental features	*Characterization of kinetic rate and equilibrium binding constants obtained from binding curves (10-min or 500-s association period and 45-min dissociation period) using the 1:1 Langmuir kinetic model*
Data source location	*Department of Immune Modulation and Biotherapeutics Discovery, Boehringer Ingelheim Pharmaceuticals, Inc., Ridgefield, CT 06877*
Data accessibility	*Data are within this article*

**Value of the data**

• The data enable the head-to-head comparison of four instruments’ performance regarding data consistency and comparability.
• The data can help new biosensor users determine the best instrument for their research purposes and maximize the value of their investment.
• The data provide additional insights for current biosensor users regarding the systematic factors that influence data reliability.

## 1 Data

Table 1 contains the calculated antibody surface binding activity for the antigen obtained using three instruments (ProteOn XPR36, Biacore T100, and Octet RED384), and Table 2, Table 3 contain the association (*k*_*a*_), dissociation (*k*_*d*_), and equilibrium (*K*_*D*_) binding constants obtained by applying the binding curves recorded using the same three instruments and the IBIS MX96. Table 4 contains the final kinetic rates and binding constants obtained from global analysis of the binding curves from multiple antibody-coated surfaces, and Table 5 contains the ratio of the instrumental limits provided by the manufacturers to the experimentally determined rate constants.

## 2 Experimental design, materials and methods

### 2.1 Proteins and antibodies

To prepare the antigen, suspended HEK293-6E cells were transfected with a plasmid encoding C-terminally 6-His-tagged human PCSK9, using the TransIT-PRO system (Mirus Bio LLC). After a 4-day incubation, the medium was harvested, and the 6-His-tagged PCSK9 was purified using a Ni-NTA His-Bind Superflow column (Novagen), according to the manufacturer׳s instructions.

To prepare 20 different monoclonal antibodies (mAbs) against human PCSK9, CHO cells were transfected with plasmid DNAs containing heavy chain and light chain cassettes, using Freestyle CHO Expression Medium containing 8 mM Glutamax (Invitrogen). After a 7-day incubation, the medium was harvested, and antibodies were purified using an ÄKTA affinity chromatography system with MabSelect Sure resin (GE Healthcare), by standard procedures [2]. The purified mAbs were formulated in 60 mM sodium acetate (pH 5.0), and their concentration was determined by the adsorption at 280 nm, using a NanoDrop™ 8000 Spectrophotometer (Thermo Fischer Scientific) and an extinction coefficient of 1.36 [2]. The antibodies were determined to be >95% monomers by size exclusion ultra-performance liquid chromatography (UPLC) (ACQUITY, Waters Corporation).

### 2.2 Instruments and reagents

Four biosensors were used for the binding experiments: the Biacore T100 [3], ProteOn XPR36 [4], Octet RED384 [5], and IBIS MX96 [6]. The Biacore T100, equipped with CM5 sensor chips, was purchased from GE Healthcare (Piscataway, NJ, USA); the ProteOn XPR36, equipped with a GLM sensor chip, from Bio-Rad (Hercules, CA, USA); the Octet RED384, equipped with AHC (anti-Human IgG Fc capture*)* biosensor tips, from ForteBio (Menlo Park, CA, USA); and the IBIS MX96, supplied with a CFM printer and SensEye COOH-G chip, from Wasatch Microfluidics (Salt Lake City, UT, USA). Recombinant protein A/G was from Thermo Fischer Scientific (catalog #21186). To couple the protein A/G to the biosensor surface, an amine coupling kit containing 10 mM sodium acetate (pH 4.5), 200 mM 1-ethyl-3-(3-dimethylaminopropyl)carbodiimide hydrochloride (EDC), 50 mM *N*-hydroxysuccinimide (NHS), and 1 M ethanolamine-HCl (pH 8.5) (GE Healthcare, product #BR1000050) or the ProteOn amine coupling kit, containing 400 mM EDC, 100 mM *N-*hydroxysulfosuccinimide (sulfo-NHS), and 1 M ethanolamine-HCl (pH 8.5) (Bio-Rad, catalog #1762410) was used. Various regeneration solutions were tested, including 4 M MgCl_2_, 10 mM glycine-HCl (pH 2.5, pH 2.0, and pH 1.5) (GE Healthcare), and 0.85% phosphoric acid (Bio-Rad) stock diluted 1/500 (v/v) in water.

### 2.3 Biacore T100 kinetic measurements

Protein A/G was immobilized onto the four individual flow cells in the CM5 sensor chip by a standard coupling protocol. First, the carboxyl groups on the sensor surface were activated by injecting 200 mM EDC/50 mM NHS. Next, protein A/G (30 µg/ml in sodium acetate [pH 4.5]) was injected over the activated surface, to which it became covalently attached by its primary amines. The excess reactive esters were then blocked with 1 M ethanolamine. Each step was performed with a 7-min injection and a 5 µl/min flow rate. The flow rate was increased to 10 µl/min for the subsequent antibody-capturing step,. Each mAb, prepared at 0.063 µg/ml in HBS-EP running buffer (10 mM HEPES [pH 7.4], 150 mM NaCl, 3 mM EDTA, and 0.005% v/v polysorbate P20) was serially injected onto flow cells 2, 3, and 4 for 220 s, 110 s, and 55 s, respectively. Flow cell 1 was left empty to provide a reference surface. To measure the binding kinetics, human PCSK9 (100–0.39 nM in 2-fold serial dilutions), and a buffer blank for baseline subtraction were sequentially injected, with a regeneration step inserted between each cycle. The protein A/G surface was regenerated with two 18-s pulses of glycine (pH 1.5) at 50 µl/min. The binding interactions were monitored over a 10-min association period and a 45-min dissociation period (running buffer only), at 30 µl/min.

### 2.4 ProteOn XPR36 kinetic measurements

To immobilize protein A/G onto the GLM sensor chip imprinted with 6 crisscrossing flow channels, a procedure similar to the above-described coupling method was used, except that 100 mM sulfo-NHS/400 mM EDC was used in the activation step. The activation, immobilization, and deactivation steps were each performed for 5 min with 6 parallel injections in the horizontal direction at 30 µl/min. The protein A/G-bound surfaces were then conditioned with three 18-s pulses of glycine (pH 1.5) at 100 µl/min in the horizontal and vertical directions. Two different mAbs, each prepared at 0.25 µg/ml, 0.125 µg/ml, and 0.063 µg/ml in PBS-T-EDTA running buffer (PBS [pH 7.4], 0.005% Tween-20, and 3 mM EDTA) were then injected in parallel in the vertical direction for 160 s at 25 µl/min. One mAb was injected into channels 1–3, and the other into channels 4–6. The orientation of the sensor chip was then switched, and a buffer blank was injected for 60 s. The antigen-binding kinetics of the mAbs in the six samples was measured by simultaneously injecting five human PCSK9 samples at different concentrations in running buffer and a buffer blank. Three different series of human PCSK9 concentration ranges (100–6.25 nM, 25–1.56 nM, and 5–0.313 nM), prepared by 2-fold serial dilution, were used. The binding was monitored over a 10-min association period and a 45-min dissociation period (running buffer only) at 40 µl/min. After each binding cycle, the protein A/G surface was regenerated with two 18-s pulses of glycine (pH 1.5) at 100 µl/min in the horizontal and vertical directions.

### 2.5 Octet RED384 kinetic measurements

Each mAb was prepared at 20 µg/ml, 10 µg/ml, and 5 µg/ml in 1×KB running buffer (PBS pH [7.4], 0.02% Tween-20, 0.1% albumin, and 0.05% sodium azide) and dispensed into a 384-well tilted-bottom microplate (90 µl per well). Eight vertical wells were used for each concentration. A second 384-well microplate contained human PCSK9 at 7 different concentrations (100–1.56 nM, in 2-fold serial dilutions), the glycine [pH 1.5] regeneration solution, and 1×KB buffer for baseline stabilization. Both plates were agitated at 1000 rpm during the entire experiment. Sixteen AHC (anti-human Fc capture) sensor tips were used for a group of 2 mAbs (8 sensors each) per binding cycle. Before the binding measurements, the sensor tips were pre-hydrated in 1×KB for 5 min, followed by 3 pre-conditioning cycles consisting of 15-s dips in glycine (pH 1.5) alternating with 15-s dips in 1×KB. The sensor tips were then transferred to the mAb-containing wells for a 200-s loading step. After a 60-s baseline dip in 1×KB, the binding kinetics were measured by dipping the mAb-coated sensors into the wells containing human PCSK9 at various concentrations. The binding interactions were monitored over a 500-s association period, followed by a 30-min dissociation period, in which the sensors were dipped into new wells containing 1×KB buffer only. The AHC sensor tips were regenerated with two 18-s dips in glycine (pH 1.5) between each binding cycle.

### 2.6 IBIS MX96 kinetic measurements

#### 2.6.1 Multi-cycle kinetics with amine-coupled antibody arrays

For multi-array printing with the CFM, two 96-well microplates were prepared. The sample source plate contained 8 vertical wells of each mAb in sodium acetate (pH 5.0) at concentrations ranging from 20 µg/ml to 0.16 µg/ml in 2-fold serial dilutions, and the reagent plate contained freshly prepared 400 mM EDC/100 mM sulfo-NHS. The COOH-G SensEye chip in the CFM was primed with sodium acetate (pH 5.0) running buffer, the sensor surface was then activated with the EDC/sulfo-NHS for 5 min, and the mAbs were then directly immobilized onto the activated surface. In the immobilization step, the mAb samples in the top half of the source plate were delivered to the sensor using 48 micro-channels, by which the mAbs were cycled across the activated surface bidirectionally for 10 min. The procedure was repeated for the remaining mAb samples in the bottom half of the source plate, generating a 10×8 array of mAb spots on the sensor surface. Two vertical columns of buffer-containing wells served as reference samples. The printed sensor chip was then inserted into the MX96 instrument and primed with the system running buffer (PBS [pH 7.4], 0.01% Tween-20). The surfaces were then quenched with 1 M ethanolamine for 5 min. For the binding measurements, human PCSK9 at 9 different concentrations (0.39–100 nM in 2-fold serial dilutions) in running buffer was cycled across the mAb array surface. Each sequentially injected sample was monitored for a 10-min association period followed by a 45-min dissociation period, at a flow rate of 40 µl/min. The amine-coupled mAb surfaces were regenerated between each binding cycle with glycine (pH 2.0 or pH 2.5). These regeneration conditions were determined in a preliminary experiment.

#### 2.6.2 Single-cycle kinetics with Fc-captured antibody arrays

A SensEye COOH-G chip was inserted into the MX96 instrument and primed with sodium acetate (pH 5.0). The sensor surface was activated by injecting 400 mM EDC/100 mM sulfo-NHS for 5 min, and then 50 µg/ml protein A/G in sodium acetate (pH 5.0) was cycled bidirectionally across the activated surface for 5 min. The sensor chip with the immobilized protein A/G surface was then removed from the MX96 and inserted into the CFM printer, which had been loaded with a 96-well mAb source plate. The same plate layout and mAb concentrations described above for the amine-coupled antibody array were used, but the mAbs were prepared in a system running buffer consisting of 0.01% Tween-20 in PBS. The mAb samples were captured by cycling them across the protein A/G surface for 10 min, then the sensor chip was inserted back into the MX96 instrument and primed with the system running buffer. The binding was measured by cycling human PCSK9 prepared at 7 different concentrations (1.56–100 nM in 2-fold serial dilutions) in the system running buffer. The cycling of each sequentially injected sample was monitored as described in the previous section. No regeneration was performed between sample injections.

### 2.7 Data analysis

#### 2.7.1 Data processing and curve fitting

The binding sensorgrams were all collected at 25 °C. Before the curve-fitting analysis, the acquired data were processed as follows. The Biacore T100 data were double-referenced using reference flow cell 1 and the subtraction of a preceding buffer blank with BiaEvaluation (v.4.1), and the ProteOn XPR36 data were double-referenced using channel inter-spots and subtraction of a parallel in-line buffer blank by the integrated ProteOn Manager software (v.3.1.0.6). The Octet RED384 data were referenced by subtracting a parallel buffer blank, and the baseline was aligned with the y-axis and smoothed by a Savitzky–Golay filter in the data analysis software (v.9.0.0.4). The IBIS MX96 data underwent inter-spot reference subtraction, followed by *y*-axis alignment using the IBIS SPRint software (v.6.15.2.1). The calibrated data were then exported to Scrubber (v.2.0c) for cropping, aligning, and buffer referencing.

The processed binding curves from the four instruments were all fitted to the Langmuir model for a 1:1 binding stoichiometry. In the Biacore T100, “single mode” was used for the fitting of data from individual mAb surfaces, and “batch mode” with “local” *R_max_* was used for the global fitting of data from multiple mAb surfaces. In the ProteOn XPR36, the “grouped” and “global” modes and the “local” *R_max_* were used for the fitting of data from single vs. multiple surfaces. In the Octet RED384, “*R_max_* linked” was used for the group fitting of data from sensors coated with the same mAb concentration, and “*R_max_* unlinked by sensor” was used for the global fitting of data from sensors coated with multiple mAb concentrations. Both the multi-cycle and single-cycle kinetic data from the IBIS MX96 were analyzed using Scrubber (v.2.0c). The *k*_*d*_ was first determined by fitting the data in the absence of *k*_a_, then the *k*_*a*_*k*_*d*_ was determined keeping the *k*_*d*_ fixed. The fit was then further refined by floating the *k*_*d*_. For single-cycle kinetic data, the injection start time was set as a floating parameter, and the association profiles were fit back to a theoretical baseline origin. In all of the analyses, *k*_*a*_ is the association rate constant for the antibody–antigen binding reaction, *k*_*d*_ is the dissociation rate constant for the antibody–antigen complex, and *K_D_* is the equilibrium dissociation constant defined by *k*_*d*_/*k*_*a*_. The fitting accuracy was described by Chi^2^ (Biacore T100, ProteOn XPR36, and IBIS MX96) or *X*^2^ (Octet RED384), a parameter representing how well the observed results resemble those calculated from the model used to analyze the data.

#### 2.7.2 Ligand surface activity

The binding activity of surface-bound mAbs toward human PCSK9, called the % ligand activity, was calculated using the following equations:

$$\%\;\mathrm{Ligand}\;\mathrm{Activity}=\frac{\mathrm{Experimental}\;R_{\max}}{\mathrm{Theoretical}{\;R}_{\max}}\times 100\%$$

where the theoretical *R_max_* (the binding capacity of the surface) was determined as follows:

$$\mathrm{Theoretical}\;R_{\max}=\frac{\mathrm{Analyte}\;\mathrm{MW}}{\mathrm{Ligand}\;\mathrm{MW}}\times R_{L}\times S_{M}$$

where MW was the molecular weight of the ligand (mAb, 150 kDa) and analyte (human PCSK9, 72.8 kDa), *R_L_* (ligand response) was the amount of immobilized ligand in response units (RU), and *S_M_* was the stoichiometry defined by the number of binding sites on the ligand. Rearranging the equation provides the calculation for the ligand density to aim for in the experiments:

$$R_{L}=\frac{\mathrm{Ligand}\;\mathrm{MW}}{\mathrm{Analyte}\;\mathrm{MW}}\times R_{\max}\times \frac{1}{S_{M}}$$

For the kinetic binding measurements, *R_max_* was set at 50–200 RU.

## Figures and Tables

**Table 1:** Antibody binding activities for human PCSK9. The slope and *R*^2^ values were obtained from the linear regression fit of the experimental *R_max_* vs. *R_L_* correlation curves (see Fig. 4 in Ref. [1]). The % activity was the ratio of the experimentally obtained slope to the theoretical *R_max_*/*R_L_* value, as defined by the known molar mass of the antigen and antibody (see Materials and methods).

**mAb ID**	**ProteOn XPR36**			**Biacore T100**			**Octet RED384**		
	**Slope**	***R***^**2**^	**% Activity**	**Slope**	***R***^**2**^	**% Activity**	**Slope**	***R***^**2**^	**% Activity**
mAb 1	0.6859	0.9986	71%	0.7938	0.9983	82%	0.6923	0.9985	71%
mAb 2	0.7368	0.9963	76%	0.7463	0.9996	77%	0.9692	0.9881	100%
mAb 3	0.7079	0.9994	73%	0.8399	0.9989	87%	0.4684	0.9763	48%
mAb 4	0.5793	0.9994	60%	0.6009	1.0000	62%	0.6000	0.9838	62%
mAb 5	0.6035	0.9999	62%	0.7056	0.9988	73%	0.6333	0.9774	65%
mAb 6	0.5595	1.0000	58%	0.9440	0.9969	97%	0.7643	0.9997	79%
mAb 7	0.7495	0.9787	77%	0.7653	0.9998	79%	1.0000	1.0000	103%
mAb 8	0.5097	0.9897	53%	0.8446	0.9999	87%	0.5214	0.9844	54%
mAb 9	0.4363	0.9743	45%	0.8488	1.0000	88%	0.8769	0.9918	90%
mAb 10	0.5653	1.0000	58%	0.7663	0.9999	79%	0.9538	0.9727	98%
**Mean**	**0.613**	**0.994**	**63%**	**0.786**	**0.999**	**81%**	**0.748**	**0.987**	**77%**
St dev	0.104	0.010	0.107	0.093	0.001	0.096	0.194	0.010	0.200
% CV	17%	0.97%	17%	12%	0.10%	12%	26%	1.02%	26%

**Table 2:** Kinetic rates and equilibrium binding constants obtained by fitting the data generated for individual antibody surfaces in the Biacore T100, ProteOn XPR36, and Octet RED384 to the 1:1 interaction model (see Figs. 7–9 in Ref. [1]).

**mAb ID**	**Surface density**	**Biacore T100**				**ProteOn XPR36**				**Octet RED384**			
		***k**_**a**_***(M**^**−1**^** s**^**−1**^**)**	***k**_**d**_***(s**^**−1**^**)**	***K**_**D**_***(M)**	**Chi**^**2**^**(RU)**	***k**_**a**_***(M**^**−1**^** s**^**−1**^**)**	***k**_**d**_***(s**^**−1**^**)**	***K**_**D**_***(M)**	**Chi**^**2**^**(RU)**	***k**_**a**_***(M**^**−1**^** s**^**−1**^**)**	***k**_**d**_***(s**^**−1**^**)**	***K**_**D**_***(M)**	***X***^**2**^**(nm)**
mAb 1	High	18.6×10^5^	3.03×10^−5^	0.016	0.1	21.7×10^5^	4.88×10^−5^	0.023	3.3	3.63×10^5^	1.59×10^−5^	0.044	2.1
	Medium	19.4×10^5^	3.16×10^−5^	0.016	0.1	21.2×10^5^	5.42×10^−5^	0.026	3.8	4.52×10^5^	1.16×10^−5^	0.026	1.8
	Low	18.6×10^5^	3.03×10^−5^	0.016	0.1	23.3×10^5^	6.38×10^−5^	0.027	2.8	5.30×10^5^	1.28×10^−5^	0.024	2.3

mAb 2	High	1.82×10^5^	2.17×10^−5^	0.120	1.1	1.23×10^5^	3.24×10^−5^	0.263	4.0	1.35×10^5^	0.63×10^−5^	0.047	2.0
	Medium	1.87×10^5^	2.85×10^−5^	0.152	0.4	1.20×10^5^	3.56×10^−5^	0.297	4.0	1.46×10^5^	0.58×10^−5^	0.040	2.2
	Low	2.00×10^5^	3.73×10^−5^	0.187	0.1	1.29×10^5^	4.71×10^−5^	0.365	3.4	1.64×10^5^	0.12×10^−5^	0.007	1.4

mAb 3	High	21.3×10^5^	25.1×10^−5^	0.118	1.5	20.1×10^5^	35.2×10^−5^	0.175	4.9	3.67×10^5^	13.1×10^−5^	0.357	4.5
	Medium	19.3×10^5^	23.8×10^−5^	0.123	0.5	20.0×10^5^	35.1×10^−5^	0.176	3.6	4.29×10^5^	12.6×10^−5^	0.294	4.5
	Low	19.6×10^5^	22.7×10^−5^	0.116	0.4	20.1×10^5^	41.0×10^−5^	0.204	2.8	5.86×10^5^	16.4×10^−5^	0.279	4.3

mAb 4	High	7.17×10^5^	24.6×10^−5^	0.344	9.8	4.81×10^5^	23.9×10^−5^	0.497	11.5	3.22×10^5^	12.6×10^−5^	0.391	7.2
	Medium	7.76×10^5^	25.2×10^−5^	0.325	2.9	4.68×10^5^	23.2×10^−5^	0.496	9.2	3.54×10^5^	12.0×10^−5^	0.338	6.5
	Low	6.80×10^5^	22.5×10^−5^	0.331	0.8	5.08×10^5^	22.1×10^−5^	0.435	5.0	4.52×10^5^	12.4×10^−5^	0.275	6.2

mAb 5	High	0.73×10^5^	3.89×10^−5^	0.535	0.8	0.61×10^5^	6.75×10^−5^	1.110	3.7	0.80×10^5^	1.61×10^−5^	0.202	1.2
	Medium	0.71×10^5^	3.70×10^−5^	0.523	0.3	0.58×10^5^	7.29×10^−5^	1.260	3.1	0.86×10^5^	0.76×10^−5^	0.088	1.4
	Low	0.87×10^5^	4.25×10^−5^	0.489	0.1	0.59×10^5^	11.6×10^−5^	1.670	3.0	0.91×10^5^	0.91×10^−5^	0.099	1.1

mAb 6	High	2.23×10^5^	7.26×10^−5^	0.326	0.8	71.2×10^5^	6.48×10^−5^	0.910	4.8	1.79×10^5^	1.68×10^−5^	0.094	1.3
	Medium	2.29×10^5^	7.16×10^−5^	0.312	0.3	70.9×10^5^	6.27×10^−5^	0.884	3.4	1.88×10^5^	3.87×10^−5^	0.206	1.4
	Low	2.46×10^5^	7.61×10^−5^	0.309	0.2	79.8×10^5^	7.03×10^−5^	0.881	3.3	1.89×10^5^	3.03×10^−5^	0.160	2.0

mAb 7	High	1.72×10^5^	14.3×10^−5^	0.828	1.3	1.04×10^5^	14.7×10^−5^	1.410	4.1	1.58×10^5^	12.1×10^−5^	0.766	1.6
	Medium	1.83×10^5^	14.1×10^−5^	0.770	0.4	1.04×10^5^	14.9×10^−5^	1.430	3.5	1.76×10^5^	11.5×10^−5^	0.652	1.9
	Low	1.94×10^5^	14.2×10^−5^	0.730	0.1	1.25×10^5^	23.4×10^−5^	1.870	3.6	1.73×10^5^	13.3×10^−5^	0.768	1.5

mAb 8	High	4.43×10^5^	19.7×10^−5^	0.444	6.4	7.57×10^5^	34.1×10^−5^	0.450	6.3	3.24×10^5^	4.29×10^−5^	0.132	2.0
	Medium	4.98×10^5^	19.6×10^−5^	0.394	1.7	7.05×10^5^	35.2×10^−5^	0.499	4.1	3.62×10^5^	5.23×10^−5^	0.144	2.4
	Low	4.90×10^5^	18.2×10^−5^	0.371	0.5	6.69×10^5^	42.3×10^−5^	0.632	3.2	4.61×10^5^	7.58×10^−5^	0.164	2.0

mAb 9	High	6.37×10^5^	5.78×10^−5^	0.091	1.8	2.20×10^5^	8.43×10^−5^	0.383	4.0	3.00×10^5^	1.08×10^−5^	0.036	2.0
	Medium	6.87×10^5^	5.93×10^−5^	0.086	0.5	2.17×10^5^	9.21×10^−5^	0.424	3.3	3.45×10^5^	0.36×10^−5^	0.010	2.9
	Low	7.03×10^5^	6.25×10^−5^	0.089	0.1	2.44×10^5^	11.2×10^−5^	0.459	2.9	4.73×10^5^	0.62×10^−5^	0.013	2.5

mAb 10	High	1.51×10^5^	9.40×10^−5^	0.624	1.0	1.04×10^5^	14.7×10^−5^	1.410	4.5	1.63×10^5^	4.09×10^−5^	0.251	2.1
	Medium	1.59×10^5^	9.68×10^−5^	0.608	0.4	1.04×10^5^	14.9×10^−5^	1.430	3.9	1.66×10^5^	3.77×10^−5^	0.227	2.0
	Low	1.66×10^5^	9.97×10^−5^	0.602	0.1	1.25×10^5^	23.4×10^−5^	1.870	3.5	1.96×10^5^	5.16×10^−5^	0.263	1.3

**Table 3:** Kinetic rates and equilibrium binding constants obtained by fitting data generated for individual antibody surfaces in the IBIS MX96 to the 1:1 interaction model (see Fig. 10 in Ref. [1]).

**mAb ID**	**Array spot**	**IBIS MX96 (amine-coupled)**				**IBIS MX96 (Fc-captured)**			
		***k**_**a**_***(M**^**−1**^** s**^**−1**^**)**	***k**_**d**_***(s**^**−1**^**)**	***K**_**D**_***(M)**	**Chi**^**2**^**(RU)**	***k**_**a**_***(M**^**−1**^** s**^**−1**^**)**	***k**_**d**_***(s**^**−1**^**)**	***K**_**D**_***(M)**	**Chi**^**2**^**(RU)**
mAb 1	1	–	–	–	–	–	–	–	–
	13	6.37×10^5^	12.3×10^−5^	0.193	2.1	3.82×10^5^	3.12×10^−5^	0.082	5.3
	25	7.51×10^5^	18.0×10^−5^	0.239	0.9	–	–	–	–
	37	6.38×10^5^	10.3×10^−5^	0.161	1.1	4.97×10^5^	3.79×10^−5^	0.076	5.0
	49	–	–	–		–	–	–	–
	61	10.0×10^5^	9.03×10^−5^	0.090	2.2	4.87×10^5^	2.92×10^−5^	0.060	3.0
	73	15.3×10^5^	9.79×10^−5^	0.064	1.1	6.56×10^5^	3.65×10^−5^	0.056	2.9
	85	–	–	–	–	–	–	–	–
									
mAb 2	2	–	–	–	–	1.26×10^5^	0.10×10^−5^	0.008	3.4
	14	–	–	–	–	1.24×10^5^	0.10×10^−5^	0.008	3.6
	26	–	–	–	–	–	–	–	–
	38	–	–	–	–	3.11×10^5^	0.10×10^−5^	0.003	4.3
	50	1.14×10^5^	0.55×10^−5^	0.048	1.3	1.63×10^5^	0.10×10^−5^	0.006	0.9
	62	1.39×10^5^	0.37×10^−5^	0.027	1.6	1.59×10^5^	0.10×10^−5^	0.006	1.8
	74	1.24×10^5^	0.42×10^−5^	0.034	1.1	1.57×10^5^	0.10×10^−5^	0.006	1.7
	86	–	–	–	–	2.09×10^5^	0.10×10^−5^	0.005	1.2
									
mAb 3	3	7.25×10^5^	33.3×10^−5^	0.460	3.1	3.44×10^5^	21.4×10^−5^	0.621	11.6
	15	5.70×10^5^	41.4×10^−5^	0.727	3.0	3.14×10^5^	21.1×10^−5^	0.671	13.5
	27	5.85×10^5^	54.6×10^−5^	0.933	1.4	6.96×10^5^	31.9×10^−5^	0.458	6.8
	39	7.14×10^5^	39.2×10^−5^	0.549	1.1	–	–	–	–
	51	9.21×10^5^	36.0×10^−5^	0.391	3.0	4.33×10^5^	20.5×10^−5^	0.474	11.4
	63	–	–	–	–	4.09×10^5^	21.7×10^−5^	0.531	9.2
	75	–	–	–	–	6.47×10^5^	27.9×10^−5^	0.432	5.2
	87	–	–	–	–	14.0×10^5^	37.9×10^−5^	0.283	1.1
									
mAb 4	4	2.05×10^5^	34.7×10^−5^	1.700	4.7	1.53×10^5^	9.19×10^−5^	0.599	10.2
	16	2.02×10^5^	39.6×10^−5^	1.960	3.5	1.39×10^5^	8.66×10^−5^	0.621	8.8
	28	–	–	–	–	–	–	–	–
	40	–	–	–	–	–	–	–	–
	52	2.56×10^5^	35.0×10^−5^	1.370	8.7	1.42×10^5^	8.85×10^−5^	0.624	9.6
	64	2.93×10^5^	37.4×10^−5^	1.280	4.4	1.62×10^5^	8.88×10^−5^	0.549	10.6
	76	2.38×10^5^	44.2×10^−5^	1.850	3.2	1.49×10^5^	12.0×10^−5^	0.804	2.0
	88	–	–	–	–	–	–	–	–
									
mAb 5	5	0.44×10^5^	4.00×10^−5^	0.901	3.3	0.31×10^5^	1.74×10^−5^	0.570	2.6
	17	0.37×10^5^	5.62×10^−5^	1.510	2.6	0.33×10^5^	1.51×10^−5^	0.453	3.5
	29	–	–	–	–	–	–	–	–
	41	–	–	–	–	0.39×10^5^	1.15×10^−5^	0.292	2.8
	53	0.38×10^5^	3.08×10^−5^	0.819	3.0	0.33×10^5^	2.05×10^−5^	0.618	2.9
	65	–	–	–	–	0.34×10^5^	2.20×10^−5^	0.656	1.9
	77	–	–	–	–	0.42×10^5^	1.76×10^−5^	0.416	1.0
	89	–	–	–	–	0.56×10^5^	2.09×10^−5^	0.375	1.0
									
mAb 6	7	0.84×10^5^	14.2×10^−5^	1.690	2.6	–	–	–	–
	19	0.63×10^5^	16.4×10^−5^	2.600	3.2	0.35×10^5^	6.21×10^−5^	1.760	5.0
	31	0.79×10^5^	13.7×10^−5^	1.740	3.6	–	–	–	–
	43	–	–	–	–	–	–	–	–
	55	0.85×10^5^	13.5×10^−5^	1.600	2.6	0.36×10^5^	5.42×10^−5^	1.490	3.3
	67	1.06×10^5^	13.2×10^−5^	1.240	1.4	0.39×10^5^	5.46×10^−5^	1.390	3.1
	79	1.00×10^5^	9.91×10^−5^	0.989	0.9	0.41×10^5^	5.57×10^−5^	1.370	2.0
	91	–	–	–	–	0.55×10^5^	13.8×10^−5^	2.520	1.1
									
mAb 7	8	1.20×10^5^	22.2×10^−5^	1.840	0.9	0.59×10^5^	18.7×10^−5^	3.170	4.1
	20	–	–	–	–	0.59×10^5^	15.7×10^−5^	2.670	6.1
	32	–	–	–	–	–	–	–	–
	44	–	–	–	–	0.28×10^5^	16.4×10^−5^	5.800	6.0
	56	1.41×10^5^	25.6×10^−5^	1.820	1.8	0.53×10^5^	17.2×10^−5^	3.280	4.5
	68	1.13×10^5^	28.9×10^−5^	2.560	2.3	0.62×10^5^	16.3×10^−5^	2.630	5.4
	80	1.52×10^5^	24.8×10^−5^	1.630	3.1	0.66×10^5^	17.4×10^−5^	2.680	2.0
	92	–	–	–	–	–	–	–	–
									
mAb 8	9	3.68×10^5^	23.5×10^−5^	0.638	6.1	1.12×10^5^	22.8×10^−5^	2.030	8.6
	21	3.70×10^5^	25.1×10^−5^	0.678	5.2	1.32×10^5^	22.4×10^−5^	1.700	6.6
	33	3.92×10^5^	32.0×10^−5^	0.815	3.1	1.03×10^5^	22.7×10^−5^	2.200	5.5
	45	3.20×10^5^	36.6×10^−5^	1.140	2.1	–	–	–	–
	57	3.78×10^5^	25.0×10^−5^	0.662	6.5	1.10×10^5^	22.1×10^−5^	2.000	7.2
	69	3.99×10^5^	31.4×10^−5^	0.784	4.2	1.20×10^5^	22.6×10^−5^	1.880	6.5
	81	3.46×10^5^	33.9×10^−5^	0.978	5.1	1.41×10^5^	25.5×10^−5^	1.820	4.8
	93	–	–	–	–	–	–	–	–
									
mAb 9	10	4.72×10^5^	11.4×10^−5^	0.242	4.1	2.08×10^5^	5.61×10^−5^	0.270	5.3
	22	5.36×10^5^	13.0×10^−5^	0.242	4.0	2.76×10^5^	8.05×10^−5^	0.292	2.7
	34	–	–	–	–	–	–	–	–
	46	8.34×10^5^	12.9×10^−5^	0.155	1.8	–	–	–	–
	58	7.36×10^5^	9.78×10^−5^	0.133	2.0	1.96×10^5^	6.92×10^−5^	0.352	6.5
	70	6.05×10^5^	11.2×10^−5^	0.186	1.9	1.99×10^5^	6.68×10^−5^	0.335	5.4
	82	–	–	–	–	2.45×10^5^	7.19×10^−5^	0.294	3.9
	94	–	–	–	–	–	–	–	–
									
mAb 10	11	0.80×10^5^	18.2×10^−5^	2.270	2.6	0.37×10^5^	8.18×10^−5^	2.210	4.3
	23	0.93×10^5^	21.0×10^−5^	2.270	1.8	0.30×10^5^	6.81×10^−5^	2.290	5.7
	35	–	–	–	–	–	–	–	–
	47	–	–	–	–	–	–	–	–
	59	1.32×10^5^	16.3×10^−5^	1.240	2.8	0.51×10^5^	9.88×10^−5^	1.950	4.7
	71	0.95×10^5^	18.5×10^−5^	1.950	2.2	0.55×10^5^	9.67×10^−5^	1.760	4.9
	83	1.34×10^5^	14.4×10^−5^	1.080	1.7	0.58×10^5^	10.6×10^−5^	1.820	2.8
	95	0.94×10^5^	15.6×10^−5^	1.660	1.1	–	–	–	–

**Table 4:** Kinetic rates and equilibrium binding constants of 10 mAbs obtained by fitting global binding curve data from multiple surfaces to the 1:1 interaction model.

**mAb ID**	**Biacore T100**			**ProteOn XPR36**			**Octet RED384**			**IBIS MX96 (Fc -captured)**			**IBIS MX96 (amine-coupled)**		
	***k**_**a**_***(M**^**−1**^** s**^**−1**^**)**	***k**_**d**_***(s**^**−1**^**)**	***K**_**D**_***(M)**	***k**_**a**_***(M**^**−1**^** s**^**−1**^**)**	***k**_**d**_***(s**^**−1**^**)**	***K**_**D**_***(M)**	***k**_**a**_***(M**^**−1**^** s**^**−1**^**)**	***k**_**d**_***(s**^**−1**^**)**	***K**_**D**_***(M)**	***k**_**a**_***(M**^**−1**^** s**^**−1**^**)**	***k**_**d**_***(s**^**−1**^**)**	***K**_**D**_***(M)**	***k**_**a**_***(M**^**−1**^** s**^**−1**^**)**	***k**_**d**_***(s**^**−1**^**)**	***K**_**D**_***(M)**
mAb 1	18.1×10^5^	3.05×10^−5^	0.017	21.7×10^5^	5.14×10^−5^	0.024	4.31×10^5^	0.99×10^−5^	0.023	5.05×10^5^	3.37×10^−5^	0.067	9.11×10^5^	11.9×10^−5^	0.131
mAb 2	1.84×10^5^	2.42×10^−5^	0.131	1.23×10^5^	3.44×10^−5^	0.280	1.56×10^5^	0.10×10^−5^	0.006	1.78×10^5^	0.10×10^−5^	0.006	1.26×10^5^	0.45×10^−5^	0.036
mAb 3	20.8×10^5^	24.7×10^−5^	0.119	20.0×10^5^	35.6×10^−5^	0.178	4.54×10^5^	12.7×10^−5^	0.279	4.74×10^5^	24.1×10^−5^	0.508	7.03×10^5^	40.9×10^−5^	0.582
mAb 4	5.36×10^5^	21.3×10^−5^	0.398	4.79×10^5^	23.5×10^−5^	0.491	4.00×10^5^	8.46×10^−5^	0.211	1.49×10^5^	8.90×10^−5^	0.597	2.39×10^5^	38.2×10^−5^	1.600
mAb 5	0.73×10^5^	3.86×10^−5^	0.530	0.60×10^5^	7.04×10^−5^	1.170	0.84×10^5^	0.50×10^−5^	0.059	0.38×10^5^	1.79×10^−5^	0.471	0.38×10^5^	4.11×10^−5^	1.080
mAb 6	2.25×10^5^	7.25×10^−5^	0.322	0.72×10^5^	6.44×10^−5^	0.894	1.76×10^5^	3.21×10^−5^	0.182	0.38×10^5^	5.67×10^−5^	1.490	0.86×10^5^	13.5×10^−5^	1.570
mAb 7	1.76×10^5^	14.2×10^−5^	0.807	1.04×10^5^	15.0×10^−5^	1.440	1.80×10^5^	10.2×10^−5^	0.567	0.54×10^5^	17.0×10^−5^	3.150	1.32×10^5^	25.4×10^−5^	1.920
mAb 8	4.56×10^5^	19.4×10^−5^	0.425	7.34×10^5^	35.0×10^−5^	0.477	3.31×10^5^	7.02×10^−5^	0.212	1.20×10^5^	23.0×10^−5^	1.920	3.68×10^5^	29.4×10^−5^	0.800
mAb 9	6.52×10^5^	5.84×10^−5^	0.090	2.20×10^5^	8.82×10^−5^	0.401	3.69×10^5^	0.10×10^−5^	0.003	2.25×10^5^	6.89×10^−5^	0.306	3.18×10^5^	11.7×10^−5^	0.368
mAb 10	1.53×10^5^	9.50×10^−5^	0.621	1.22×10^5^	23.1×10^−5^	1.890	1.73×10^5^	3.72×10^−5^	0.215	0.46×10^5^	9.03×10^−5^	1.960	0.52×10^5^	17.3×10^−5^	3.320

**Table 5:** Ratio of the recommended instrumental working limits to the analyzed kinetic rate constants.

**mAb ID**	**Biacore T100**^a^		**ProteOn XPR36**^b^		**Octet RED384**^c^		**IBIS MX96**^d^		**IBIS MX96**^d^	
							**(Fc -captured)**		**(amine-coupled)**	
	***k**_**a**_*	***k**_**d**_*	***k**_**a**_*	***k**_**d**_*	***k**_**a**_*	***k**_**d**_*	***k**_**a**_*	***k**_**d**_*	***k**_**a**_*	***k**_**d**_*
mAb 1	5.5	3.1	1.4	51.4	23.2	10.0	5.5	59.5	9.9	16.9
mAb 2	54.3	2.4	24.4	34.4	64.1	1.0	39.7	2.2	28.1	0.5
mAb 3	4.8	24.7	1.5	356.0	22.0	127.0	7.1	204.5	10.5	120.5
mAb 4	18.7	21.3	6.3	235.0	25.0	84.6	20.9	191.0	33.6	44.5
mAb 5	137.4	3.9	49.9	70.4	119.0	5.0	131.9	20.6	130.5	9.0
mAb 6	44.4	7.3	41.9	64.4	56.8	32.1	58.1	67.5	131.9	28.4
mAb 7	56.8	14.2	28.8	150.0	55.6	102.0	37.9	127.0	91.9	85.0
mAb 8	21.9	19.6	4.1	350.0	30.2	70.2	13.6	148.0	41.7	115.0
mAb 9	15.3	5.8	13.6	88.2	27.1	1.0	7.9	58.5	22.2	34.5
mAb 10	65.4	9.5	24.6	231.0	57.8	37.2	48.1	86.5	108.2	45.2

a Uppermost *k*_*a*_ limit (M^−1^ s^−1^): 1×10^7^; lowest *k*_*d*_ limit (s^−1^): 1×10^−5^[7].

b Uppermost *k*_*a*_ limit (M^−1^ s^−1^): 3×10^6^; lowest *k*_*d*_ limit (s^−1^): 1×10^−6^[8].

c Uppermost *k*_*a*_ limit (M^−1^ s^−1^): 1×10^7^; lowest *k*_*d*_ limit (s^−1^): 1×10^−6^[9].

d Uppermost *k*_*a*_ limit (M^−1^ s^−1^): 5×10^6^; lowest *k*_*d*_ limit (s^−1^): 2×10^−6^[10].
